# Rapid signal enhancement method for nanoprobe-based biosensing

**DOI:** 10.1038/s41598-017-07030-0

**Published:** 2017-07-28

**Authors:** Jorge T. Dias, Gustav Svedberg, Mats Nystrand, Helene Andersson-Svahn, Jesper Gantelius

**Affiliations:** 1 Division of Proteomics and Nanobiotechnology, Science for Life Laboratory, KTH Royal Institute of Technology, Stockholm, Sweden; 2 Global Research and Development, Thermo Fisher Scientific IDD, Uppsala, Sweden

## Abstract

The introduction of nanomaterials as detection reagents has enabled improved sensitivity and facilitated detection in a variety of bioanalytical assays. However, high nanoprobe densities are typically needed for colorimetric detection and to circumvent this limitation several enhancement protocols have been reported. Nevertheless, there is currently a lack of universal, enzyme-free and versatile methods that can be readily applied to existing as well as new biosensing strategies. The novel method presented here is shown to enhance the signal of gold nanoparticles enabling visual detection of a spot containing <10 nanoparticles. Detection of Protein G on paper arrays was improved by a 100-fold amplification factor in under five minutes of assay time, using IgG-labelled gold, silver, silica and iron oxide nanoprobes. Furthermore, we show that the presented protocol can be applied to a commercial allergen microarray assay, ImmunoCAP ISAC sIgE 112, attaining a good agreement with fluorescent detection when analysing human clinical samples.

## Introduction

Biosensing applications have benefited considerably from the introduction of nanomaterials such as gold nanoparticles (AuNPs) or iron oxide nanoparticles (IONPs)^1^. Optical properties such as surface plasmon resonance in the case of AuNPs or magnetic properties of the IONPs have contributed to improved sensitivity in biodetection applications^2, 3^. More generally, the large surface to volume ratio of affinity labelled nanomaterials together with the myriad of protocols for their surface decoration with various ligands have made such materials interesting tools for the development of more efficient and sensitive sensors^4^. Nonetheless, large nanoprobe numbers are typically still required to achieve detectable signals when used as biosensing tools. For instance, in the case of 40 nm AuNPs approximately 90 million nanoparticles (NPs) were required to obtain a colorimetric UV-vis spectroscopy detection^5^. To circumvent sensitivity limitations due to low nanoprobe densities, several strategies of signal amplification have been developed^6^. Such enhancement techniques are often based on either interparticle aggregation or staining of the nanoprobe with a material that allows improved signal acquisition. The former relies on the gathering of a higher number of nanoparticles on the nanoprobe site after the detection has occurred. Increasing the density of nanoparticles allows visual signal acquisition or UV-Vis measurement^7^. The gathering of extra nanoparticles on the detection site can be achieved by targeting a second set of nanoparticles to the initial detection nanoprobes. The effectiveness of the signal enhancement will be intrinsically associated with the recognition capacity of the nanoprobes by this second set of nanoparticles.

Alternative enhancement strategies such as silver staining of either gold or silver nanoprobes^8, 9^ rely on the reduction of silver ions onto the surface of the nanoparticles creating a silver precipitation film detectable visually or by UV-Vis analysis^10^. Such techniques’ specificity is determined by the presence or absence of either AuNPs or silver nanoparticles (AgNPs), thus it is possible to apply it without the restrictions associated with an interparticle aggregation method. Thus far, silver staining techniques have only be shown to be applied to either AuNPs or AgNPs^10, 11^.

Zayats *et al*.^12^ showed in 2005 that it was possible to potentiate the surface plasmon resonance signal of AuNPs by promoting the reduction of Au ions onto the surface of existing gold nanoprobes. In their work, hydrogen peroxide generated by glucose oxidase catalysis and cetyltrimethylammonium chloride were used to achieve the reduction of the chloroauric acid. However, only enhancement of gold nanoprobes was studied, and a spectrophotometer was required for signal acquisition. Wang *et al*.^13^ reported a colorimetric-based enhancement method where a sandwich based assay was developed for detection of human IgG using AuNPs. After detection, HAuCl_4_·4H_2_O and NH_2_OH·HCl were added to the system, forming a new gold layer on the surface of the AuNPs. These enlarged AuNPs showed a peroxidase-like catalytic ability against the substrate 3,3′,5,5′-tetramethylbenzidine (TMB) and a bright blue colour was observed. With this enhancement strategy, the authors were able to detect as low as 3 × 10^−10^ g·mL^−1^ of human IgG. More recently Stevens *et al*.^14^ developed a plasmonic ELISA-based assay where AuNPs allowed ultrasensitive visual detection of a prostate-specific antigen (PSA). The detection strategy consisted of a traditional ELISA followed by the labelling of the primary antibody with a secondary antibody previously modified with catalase. The sensor was immersed in a solution that contained H_2_O_2_ and chloroauric acid. If the secondary antibody carrying catalase did not detect the primary antibody, the H_2_O_2_ in solution was not consumed and the chloroauric acid was reduced at a fast rate. In the presence of analyte and formation of the complex primary antibody – secondary antibody modified with catalase, the enzyme could consume H_2_O_2_ and so decrease the reduction kinetics of the chloroauric acid. This difference in the speed at which chloroauric acid was reduced affected the morphology of the AuNPs formed. Higher concentrations of H_2_O_2_ allowed the formation of quasi-spherical monodispersed gold nanoparticles with a red colour, lower concentrations of H_2_O_2_ yielded ill-defined aggregate-like structures that showed a blue colour. This red/blue colour dichotomy allowed the authors to detect as low as 10^−18^ g·mL^−1^ of PSA in whole serum. Similar to the work of Zayats *et al*., the enhancement effect demonstrated by Stevens *et al*. was promoted by the reduction of chloroauric acid to gold ions by hydrogen peroxide. Here, the concentration of available hydrogen peroxide was determined by the activity of catalase and subsequently correlated to the concentration of the analyte being detected. The need to modify a detection antibody with an enzyme and control of the catalytic activity are two limiting factors for the universality of the method proposed by Stevens *et al*. Furthermore, the conditions used do not account for the formation of gold clusters independently of the reporter AuNPs. This may lead to difficulty controlling the background amplification.

The above and other^15–17^ elegant strategies for signal enhancement have allowed detection limits that rival those of traditional techniques such as PCR or ELISA.

Here, we present a novel gold enhancement method that allows amplification of the signal given by a reporter nanoprobe by potentiating or introducing a surface plasmon resonance signal. The strategy is based on two physical principles described by Mie^18^ and Prodan *et al*.^19^ Mie’s theory of light scattering and absorption describes the plasmon resonances of electrons confined to nanoscale volumes, showing that the larger the particle the more efficient the scattering is in detriment of the absorption efficiency. An in-depth review on this phenomenon can be read in a review by Fan *et al*. on light scattering and surface plasmons^20^. In our system, as the seed nanoparticles grow in size by the deposition of Au(0), the amount of scattered light increases and since the frequency of the plasmon resonance remains within the visible wavelength, the signal observed by the naked eye is potentiated. There is, however, a second phenomenon at work, Prodan *et al*. proposed a new theoretical approach to Mie’s theory. It was suggested a parallel between the behaviour of plasmons in grouped metallic nanoparticles and electrons in quantum molecular orbitals. The theory states that the plasmons of neighbouring metallic nanoparticles interact as electronic wave function of atomic and molecular orbitals do. As in each spot of the arrays there are several nanoprobes after detection is carried out, the deposition of Au(0) will promote the increase in size of these nanoprobes. Thus, the plasmons of these neighbouring Au nanostructures interact, resulting in an augmented scattering of light that translates into an increase in signal intensity or the surfacing of a visible signal where none previously existed. The light scattered (signal) can be acquired by UV-Vis spectroscopy or by image acquisition using consumer-grade tools such as a tabletop scanner or digital camera. We show that this method can be applied not only to AuNPs and AgNPs but also to IONPs and silica nanoparticles (SiNPs), using a generic and enzyme-free protocol. Moreover, we demonstrate that the method can be applied successfully in a commercial allergy diagnostic ImmunoCAP ISAC sIgE 112 assay (ThermoFisher) without specific optimisation.

## Results and Discussion

**Figure 1:**
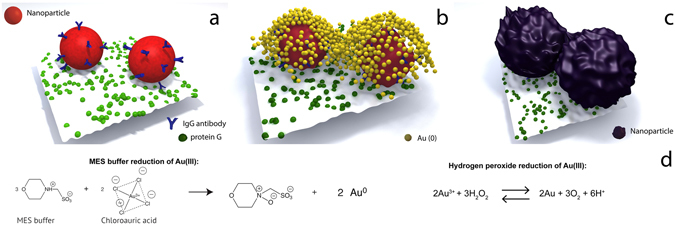
Illustration of (**a**) two neighbouring IgG-nanoprobes after detecting protein G immobilized on a microspot, (**b**) Incubation of the microspot array with enhancement solution and consequent seed-growth of the IgG-nanoprobes, (**c**) IgG-nanoprobes after 5 minutes of incubation with the enhancement solution and when the Au(0) atoms have been depleted and (**d**) chemical processes describing the reduction of Au(III) to Au(0).

The enhancement of the signal of nanoprobes after the detection of a target (Fig. 1a) here reported is based on the capability of MES buffer as well as H_2_O_2_ to reduce Au(III) to Au(0) (Fig. 1d). By optimising the concentrations of HAuCl_4_, H_2_O_2_ and MES buffer as well as reaction pH it was possible to favour the deposition of Au(0) onto existing nanoparticles (Fig. 1b) rather than the formation of new nanoparticles (see supporting information Characterisation and optimisation of enhancement solution). The growth in size of the existing seeds (nanoprobes) and their proximity in the microarray spots yields the increase in scattering light, while remaining within the visible range of the spectrum (Fig. 1c) thus, the enhancement of signal.

It has been shown that parameters such as the precursor concentration (chloroauric acid), reducing agents (MES buffer and hydrogen peroxide), electrolyte concentration, pH and temperature influence the synthesis of AuNPs, and in particular the speed at which the nucleation and formation of new nanoparticles occurs^21^. Several reports in literature have shown that tweaking these parameters allows the seed-based growth of existing nanoparticles, in the case of AuNPs, or the formation of core-shell nanoparticles where the core is composed of AgNPs^22^, SiNPs^23^ or IONPs^24^, for instance, and the shell made of a gold layer. The optimisation of our method, concentration of precursor and reducing agents (see supporting information Characterisation and optimisation of enhancement solution), was carried out with the aim of Au(0) deposition to occur preferably on the nanoprobes bound to the microarray paper or glass substrates. We observed that the signal intensity would improve when using 50 mM MES buffer and 5 mM HAuCl_4_.3H_2_O as the Au(III) source, moreover we observed that the intensity of the signal would increase with increasing values of pH of the MES buffer (see Supporting information Figure S6a–e). Additionally, we observed that when H_2_O_2_ was added to the enhancement solution the S/N ratio was improved (see Supporting information Figure S6f–j). H_2_O_2_ is also a reducing agent for Au(III) and has been shown to favour the formation of spherical gold structures^25, 26^. The formation of AuNPs with a well-defined shape is more time-consuming when compared to the formation of amorphous AuNPs^27^. Thus, the presence of H_2_O_2_ should slow down the de novo synthesis of AuNPs, further favouring the deposition of Au(0) onto the existing seeds. Although a 100x-fold improvement of the signal to noise level was achieved using an enhancement solution consisting of 5 mM HAuCl_4_·3H_2_O, 50 mM MES pH 5, 1.027 M H_2_O_2_, a time-lapse study (Figure S7) showed that the time required for signal enhancement could be improved by more than half (300 vs 120 seconds) when 10 mM MES pH 6, 1.027 M H_2_O_2_ was used. Such observation led us to use as enhancement solution a solution composed of 5 mM HAuCl_4_, 10 mM MES pH 6, 1.027 M H_2_O_2_. Henceforward when we refer to enhancement solution we are referring to a solution with these components, at these concentrations.

To evaluate the capacity of our method in revealing seemingly invisible nanoparticles to the naked eye, a dilution series from a stock AuNPs suspension was printed onto paper. The number of nanoparticles per spot ranged from ~100,000 down to <10 (see Supporting information Figure S8), including negative control spots containing only printing buffer. Prior to enhancement, only the spots with 10,000 or more AuNPs could be detected by naked eye or optical scanning. After a 5-minute incubation with the enhancement solution, all AuNPs-containing spots had increased signals. Moreover, the spots harbouring less than 10 AuNPs were weak in intensity but clearly detectable, whereas negative control spots remained with no detectable signal.

Even though the optimisation of the components of the enhancement solution was carried out using IgG-modified AuNPs, there is a multitude of assays reported in the literature making use of other types of nanoprobes, e.g. AgNPs, IONPs or SiNPs. Thus, we studied the efficiency of the method in assays that use those types of nanoprobes as detection agents.

**Figure 2:**
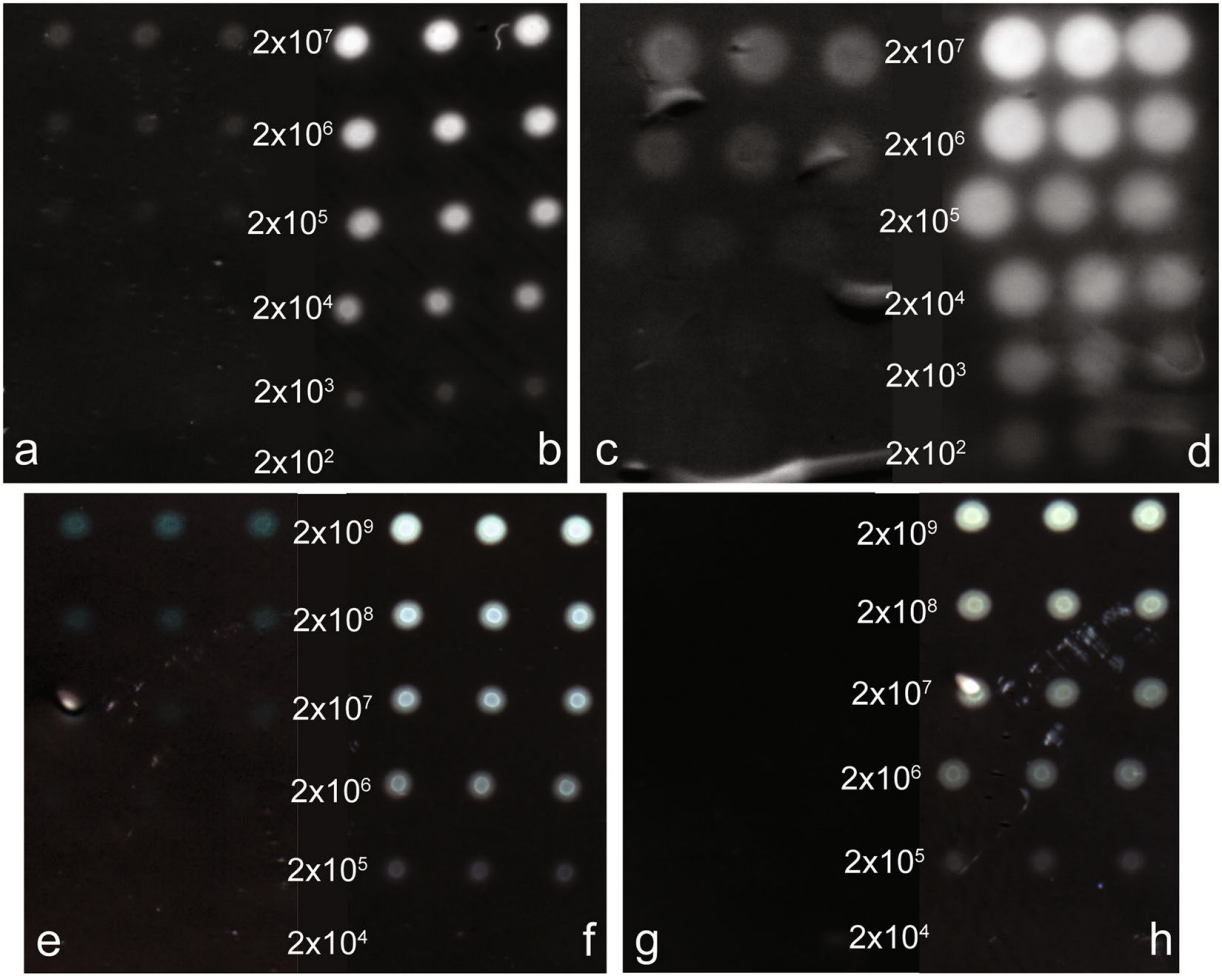
Vertical flow arrays for detection of protein G. Each concentration of protein G was printed in triplicate. (**a**) IgG-AuNPs detection prior to enhancement. (**b**) IgG-AuNPs detection after enhancement. **(c**) IgG-AgNPs detection prior to enhancement. (**d**) IgG-AgNPs detection after enhancement. (**e**) IgG-IONPs detection prior to enhancement. (**f**) IgG-IONPs detection after enhancement. (**g**) IgG-SiNPs detection prior to enhancement. (**h**) IgG-SiNPs detection after enhancement. Middle column refers to the number of Protein G molecules per spot. Approximately ¼ of the expected radius of the spot was considered for imaging analysis and protein quantification.

AuNPs, AgNPs, SiNPs and IONPs were modified with IgG (see Supporting information Characterisation of the different sets of nanoparticles) and used as reporter agent in the detection of a gradient of a number of Protein G molecules per spot (Fig. 2). The choice of sizes for the different types of NPs was done following the criteria of working with sizes common in the published literature which were deemed convenient to synthesise (in the case of AuNPs and AgNPs) and available to purchase (in the case of SiNPs and IONPs).

Except for the IgG-SiNPs, for which no signal was observed prior to enhancement (Fig. 2g), all other nanoparticles were capable of providing a visible signal pre-enhancement. Untreated arrays allowed the detection of as low as 2 × 10^5^ molecules per spot (Fig. 2a), 2 × 10^4^ molecules per spot (Fig. 2c) and 2 × 10^7^ molecules per spot (Fig. 2e) for IgG-AuNPs, IgG-AgNPs and IgG-IONPs, respectively.

After incubation with the enhancement solution, a 100-fold increase in signal was observed for all types of NPs used except for the IgG-SiNPs. For the IgG-AuNPs the spots where 2 × 10^3^ protein G molecules were printed were now visible (Fig. 2b). For the IgG-AgNPs the spots with 2 × 10^2^ protein molecules became visible (Fig. 2d) and for the IgG-IONPs the spots containing 2 × 10^5^ protein molecules became visible (Fig. 2f). Lastly, the enhancement solution made visible several of the protein G spots in the case of the SiNPs (Fig. 2h). In this case, more than an improvement factor, the enhancement solution is determinant to allow the use of SiNPs in colorimetric detection systems.

Colorimetric biosensing typically relies on the correlation between the intensity of the signal and the concentration detected to enable a quantitative analysis^28^. In this work, we show that the signal intensity for each spot concentration was observed to increase while maintaining a linear correlation to the printed analyte, and consequently, quantitative analyte estimation can be performed post-enhancement (see supporting information Table S1).

To access the applicability of our method in an existing commercial immunoassay platform, we carried out the enhancement strategy on an ImmunoCAP ISAC sIgE 112 allergen component microarray immunoassay. This commercial allergen array assay consists of a panel of 112 components from 51 allergen sources and has been described in detail^29^.

**Figure 3:**
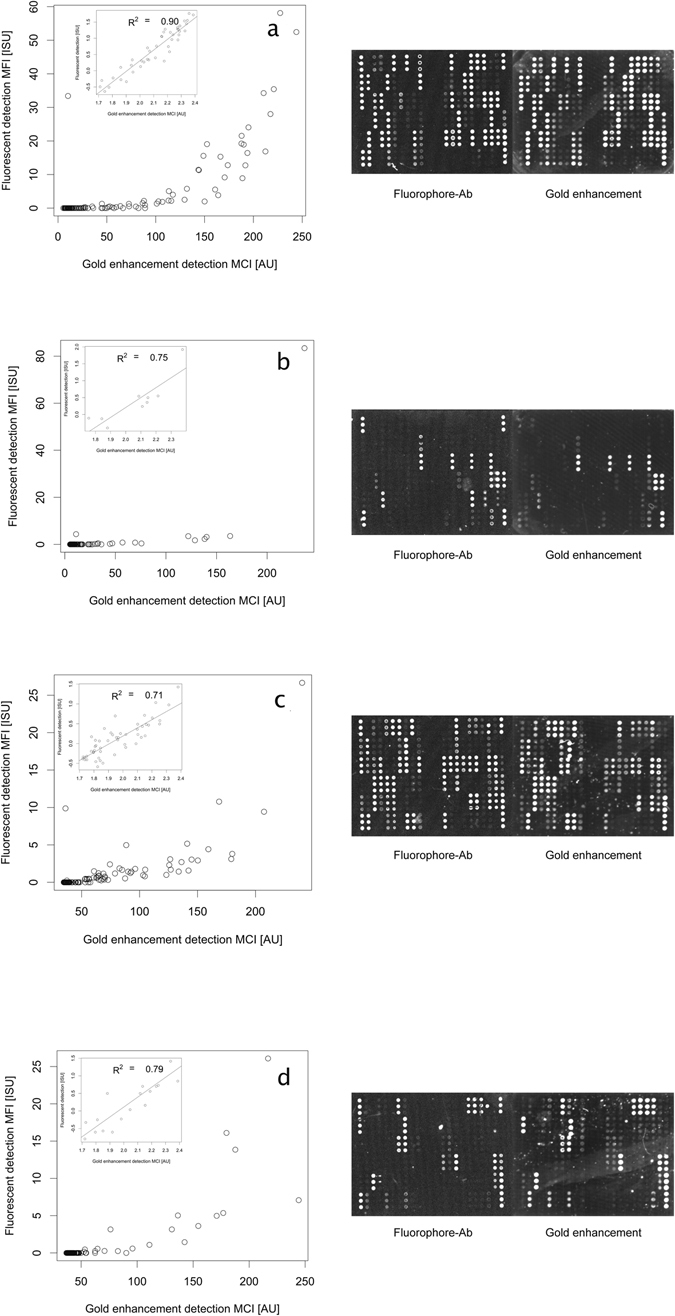
Comparison of mean fluorescent intensity (MFI) obtained with fluorescence detection and mean colorimetric intensity (MCI) obtained with gold enhancement detection for samples (**a**,**b**,**c** and **d**). Insets display the same data log 10 transformed in the interval where there is a linear correlation between the two methods. For each sample, the arrays were scanned for further data analysis. In the fluorescence-based detection, the brightness of spots is fluorescence emission, whereas in the gold enhancement-based detection, the image has been inverted and increased brightness indicates a colourimetrically darker spot. Allergens are deposited in vertical triplicates with positive controls for fluorescent detection on the far-right bottom (the remainder three corners are used for software evaluation). Images processed in 16-bit grayscale, the range of intensity levels are 0–65536.

The ImmunoCAP ISAC sIgE 112 allergen microarray was used to analyse a set of 4 validated and characterised serum samples from allergic patients (designated as samples a, b, c and d). Fluorimetric detection was compared to staining with anti-IgE labelled AuNPs followed by enhancement (Fig. 3).

On the gold enhancement-based detection assay, no spots were detected in the tabletop scanner images prior to enhancement (see Supporting information Figure S4), whereas after the enhancement procedure, a variety of spots were detectable by scanning and visible to the naked eye as well (Fig. 3). The two detection methods were compared by comparing the mean fluorescence intensity (MFI) of the standard ImmunoCAP ISAC sIgE 112 assay and the mean colorimetric intensity (MCI) of the gold enhancement-based assay. The data obtained for these 4 clinical samples showed a good concordance between the two detection methods, with an average R2 = 0.79+/−0.08 when data is 10-logged on both axes.

Interestingly, as shown in Fig. 3, there is a range of data points where the fluorimetric detection yields values that cluster close to zero, whereas in the gold enhanced setting a linear distribution was observed. Nonetheless, there are also several data points where the gold enhanced setting seems to fail detection when compared to the fluorimetric detection. The allergens are being further studied individually as to evaluate which can be detected with higher sensitivity with the gold enhanced settings. Further, any effects on nanoprobe binding and enhancement resulting from stirring and other convective disturbances, while interesting and potentially useful were outside the scope of this study.

This comparison experiment indicates that our enhancement method can be readily applied to an existing commercial kit with the added advantage of decreasing the assay total time while enabling a colorimetric detection with seemingly retained or improved sensitivity and dynamic range.

## Conclusion

Even with the best available affinity reagents, advanced sensor surfaces and efficient microfluidic mass transport of analytes and detection reagents, the high surface density of reporter molecules or materials such as fluorophores and nanoparticles typically required for detection may limit real world sensitivity a thousand-fold or more. Consequently, there is a great need for generic, rapid and robust enhancement methods that can enable ultra-sensitive biosensing assays amenable for the advanced laboratory as well as low-cost point of care tests.

In nanoparticle-based biosensing methods, the visualisation of a single nanoparticle currently requires high-end technologies such as scanning electron microscopy (SEM) or confocal microscopy. However, these technologies are currently incompatible with rapid and low-cost detection strategies. For common detection techniques, such as laser scanning for fluorimetric detection or CCD/CMOS-sensors for colorimetric readout, the high number of reporters required for a detectable signal may presently limit the overall assay sensitivity.

In this work, we present a novel convenient, generic and enzyme-free gold enhancement protocol where *in situ* growth is promoted preferentially at the location of a surface-bound metal-nanoprobe, which may enable biosensing of extremely low biomarker concentrations. The enhancement protocol appears to be versatile and could be performed in both physiological and non-physiological conditions, in assays where one or several types of metal nanoparticles were used for detection, under ambient light, immediately after the assay is performed or up to several weeks after. The amplification process could be observed by naked eye or digital camera in real time due to the enhancement solution being largely transparent and light-insensitive, and consequently, the rate of the signal could be utilised for rapid and comprehensive analysis of the results.

A 100-fold enhancement factor was observed across the different types of nanoprobes used, representing, for instance, in the case of AgNPs the detection of as low as 200 protein molecules per spot. The use of a single enhancement solution to achieve enzyme-free signal amplification that is suitable for several types of nanoprobes confers universality to the method here presented.

Proof-of-concept preliminary experiments, designed to assess the efficacy and validation of the enhancement method in pre-existing commercial immunoassays, have shown that the method can be readily applied with good concordance (R^2^ = 0.79 + / − 0.08) between the conventional fluorescence readout and our proposed colorimetric readout. Also, it can be applied readily after the assay is performed or later in time, as long as the nanoprobes are still present on the sensor surface. If the sensor is well preserved this can be up to months after the assay was carried out. The above characteristics make this protocol, to the best of our knowledge, unmatched in the field of colorimetric signal enhancement techniques.

Future work will include employing the enhancement method for detection and quantification of low abundant plasma proteins as well as rapid and sensitive genotyping of microbial infections.

## Methods

Unless stated otherwise, all chemicals are from Sigma-Aldrich and were used as received without further purification.

### Ethical statement

Human serum samples were provided by ThermoFisher® and were obtained in accordance to the legal and ethical requirements of the country of collection, i.e with the approval of an ethics committee (or similar) and with written consent from the donor.

### AuNPs and AgNPs synthesis

AuNPs of 10 nm in diameter were prepared following the protocol described by Bastús *et al*.^18^. Briefly, an aqueous HAuCl_4_ precursor was injected into a boiling solution of sodium citrate. The reaction was allowed to occur until a red-wine colour was observed. AuNPs of 40 nm in diameter were prepared following a stepwise seed growth protocol, also described in the same work. After preparing the 10 nm AuNPs seeds, the temperature of the solution was decreased to 90 °C and both sodium citrate and HAuCl_4_ precursors were injected. The protocol was followed until nanoparticles of 40 nm were obtained.

AgNPs of 90 nm in diameter were prepared following the protocol described by Rivero *et al*.^19^. Briefly, 1.6 mM dimethylaminoborane (DMAB) was added to a vigorously stirred solution which contained 10 mM poly(acrylic acid, sodium salt) (PAA) and 3.33 mM AgNO_3_. The size was determined by UV-Vis spectroscopy according to the AgNPs size theory demonstrated by Malynych^20^.

IONPs of 100 nm in diameter decorated with carboxyl groups were purchased from Ademtech, France. SiNPs of 50 nm in diameter decorated with carboxyl groups were purchased from Corposcular, USA.

### AuNPs, AgNPs, SiNPs and IONPs modification with antibodies

Prior to the functionalization of the AuNPs with antibodies, AuNPs were derivatized with COOH-PEG-SH (5000 g·mol^−1^) (Rapp Polymere GmbH). 10 mL (1 mL – 1 OD) of AuNPs were incubated with 1 mg of thiolated PEG. The pH of the solution was adjusted to 12 with a concentrated solution of NaOH. The solution was left overnight to react under mild stirring conditions. The excess of reagents was removed by centrifugation at 13800 × g for 15 minutes. The pellets were redispersed in Mili-Q water.

The synthesis of the AgNPs is achieved using a polymer (PAA) that exposes carboxyl groups to the outer surface of the nanoparticles.

The coupling of antibody to the NPs was prepared following a modified version of a protocol previously reported by Puertas *et al*.^21^. Briefly, 0.5 nM of AuNPs were allowed to incubate with 5 μmol of N-(3- Dimethylaminopropyl)-N’-ethylcarbodiimide hydrochloride (EDC) and 7.5 μmol of N- Hydroxysulfosuccinimide sodium salt (sulfo-NHS) in 10 mM 2-(N-Morpholino)ethanesulfonic acid (MES) pH 6 buffer for 30 minutes at 37 °C. After this incubation period the AuNPs were washed by centrifugation to remove the excess of EDC and sulfo-NHS as well as the byproduct formed by these two reagents (O-acylisourea). The AuNPs were incubated with 100 pg·mL^−1^ of antibody for 2 hours at 37 °C in 10 mM MES pH 5 buffer. The excess of uncoupled antibody was washed by centrifugation. The excess of non-covalent coupled antibody was removed by centrifugation after incubating the AuNPs with 10 mM sodium phosphate pH 7.5, 0.3 M NaCl buffer for 30 minutes at 37 °C.

The IgG-modified AuNPs were incubated overnight with 1% bovine serum albumin (BSA) at 4 °C in 10 mM MES pH 6 to block unreacted carboxyl groups on the surface of the NPs. Finally, the IgG-modified AuNPs were washed with 10 mM MES pH 6 buffer by centrifugation to remove the excess of BSA and stored at 4 °C for further use.

5pM of AgNPs, 0.5 mg of IONPs and  0.5 mg of SiNPs were coupled to IgG antibody following the same EDC chemistry coupling method.

### Microarray printing

Microarrays were prepared as previously reported by our group^22^. Briefly, protein G was deposited in gradient concentrations on nitrocellulose paper membrane Protran BA79 0.1 μm (Whatman) using a NanoPlotter NP 2.1 robotic printer (GeSim). The arrays were printed with 10 droplets per spot giving an approximate printing volume of 3 nL per spot. Each spot has a diameter of approximately 200 μm. After printing the membranes were allowed to dry overnight at room temperature.

### Vertical flow assays

The array membrane was placed inside a XX30001200 Swinny Filter Holder 13 mm (Merck Millipore). All IgG-modified nanoparticles were redispersed in an assay buffer that consisted of 29 mM sucrose, 0.44 mM BSA, 0.45 M NaCl, 0.5% w/v Tween 20 in 0.1 M phosphate pH 7.4 buffer. IgG-modified AuNPs were prepared as to have 8 pM, IgG-modified AgNPs were prepared as to have 17 pM, IgG-modified SiNPs were prepared as to have 0.1 mg·mL^−1^ and IgG-modified IONPs were prepared as to have 0.1 mg·mL^−1^. The flow was controlled using a PhD2000 ultrasyringe pump (Harvard Apparatus) and set to a rate of 1 mL·min^−1^. The arrays were left to dry at room temperature for 5 minutes before being digitalized in a flatbed scanner CanoScan 9000 F Mark II (Canon) in 24-bit colour.

### Enhancement protocol

After the vertical flow assay, the arrays were removed from the holder and placed in the well of a 24-well polystyrene plate (Corning). The enhancement solution was added to the well and the array allowed to incubate for up to 300 seconds. The enhancement solution’s components are described in more detail in the results and discussion section. Following incubation with the enhancement solution, the arrays were removed from the well and allowed to dry for 5 minutes at room temperature, after which were digitalized in the previously referred scanner.

### Reference Assay ImmunoCAP ISAC sIgE 112

The ImmunoCAP ISAC sIgE 112 is an *in vitro* semi-quantitative assay for the measurement of allergen-specific IgE antibodies. Allergen components are immobilised on a solid glass substrate in a microarray format. One glass slide contains 4 microarrays. The assay was performed following the manufacturer’s Direction for Use (DfU). Each microarray was incubated with one sample of human serum for 120 minutes at room temperature in order to bind sIgE, if present, to the immobilised allergen components. Subsequently, the array was rinsed with H_2_O and then incubated for 30 minutes with a fluorescence-conjugated anti-human IgE antibody. Finally, after a second wash step, the fluorescence intensity of each microarray was read by laser scanning (LuxScan 10/K, Capital Bio). The analysis of the digitalized images was performed with the software Phadia Microarray Image Analyzer, MIA 1.2.4 (ThermoFisher). This software allows transforming the fluorescence intensity in numerical data according to the calibration curve built with a calibrator sample included in each assay. Results equal or greater than 0.30 ISU were considered positive, according to the DfU of the manufacturer.

### ImmunoCAP ISAC sIgE 112 gold enhancement assay

The assay was carried out following the manufacturer’s DfU with some modifications. The array slide was allowed to incubate for 120 minutes at room temperature with a human serum sample. The slide was rinsed with H_2_O and then incubated with anti-human IgE modified AuNPs for 30 minutes at room temperature. Finally, after a third washing step, the microarray was incubated with the gold enhancement solution consisting of 5 mM HAuCl_4_, 1.027 M H_2_O_2_ in 10 mM MES pH 6 for 5 minutes. The microarray was then digitalized using a consumer-grade table-top scanner (Image Scanner Epson Expression 1600 Pro). The mean colorimetric intensity (MCI) in each spot was measured and for each set of triplicate allergens spot, the mean of this value was calculated and treated as the signal from that allergen.

## Electronic supplementary material

Rapid signal enhancement method for nanoprobe-based biosensing SI

## References

[1] Holzinger M, Le Goff A, Cosnier S (2014). Nanomaterials for biosensing applications: a review. Front. Chem..

[2] Kim C-B, Lim E-G, Shin SW, Krause HJ, Hong H (2016). Magnetic immunoassay platform based on the planar frequency mixing magnetic technique. Biosens. Bioelectron..

[3] Omidfar K, Khorsand F, Darziani Azizi M (2013). New analytical applications of gold nanoparticles as label in antibody based sensors. Biosens. Bioelectron..

[4] Conde J (2014). Revisiting 30 years of biofunctionalization and surface chemistry of inorganic nanoparticles for nanomedicine. Front. Chem..

[5] Sato K, Onoguchi M, Sato Y, Hosokawa K, Maeda M (2006). Non-cross-linking gold nanoparticle aggregation for sensitive detection of single-nucleotide polymorphisms: Optimization of the particle diameter. Anal. Biochem..

[6] Cao X, Ye Y, Liu S (2011). Gold nanoparticle-based signal amplification for biosensing. Anal. Biochem..

[7] Fraire JC, M. RD, C. EA (2016). Design of a novel plasmonic nanoconjugated analytical tool for ultrasensitive antigen quantification. Nanoscale.

[8] Taton TA (2000). Scanometric DNA Array Detection with Nanoparticle Probes. Science.

[9] Cao C, Gontard LC, Thuy Tram LL, Wolff A, Bang DD (2011). Dual Enlargement of Gold Nanoparticles: From Mechanism to Scanometric Detection of Pathogenic Bacteria. Small.

[10] Lu Y, Shi W, Qin J, Lin B (2009). Low cost, portable detection of gold nanoparticle-labeled microfluidic immunoassay with camera cell phone. Electrophoresis.

[11] Zhang M, Wittstock G, Shao Y, Girault HH (2007). Scanning electrochemical microscopy as a readout tool for protein electrophoresis. Anal. Chem..

[12] Zayats M, Baron R, Popov I, Willner I (2005). Biocatalytic Growth of Au Nanoparticles: From Mechanistic Aspects to Biosensors Design. Nano Lett..

[13] Wang S, Chen Z, Choo J, Chen L (2016). Naked-eye sensitive ELISA-like assay based on gold-enhanced peroxidase-like immunogold activity. Anal. Bioanal. Chem..

[14] la Rica deR, Stevens MM (2012). Plasmonic ELISA for the ultrasensitive detection of disease biomarkers with the naked eye. Nat. Nanotechnol..

[15] Liu D (2014). Glucose Oxidase-Catalyzed Growth of Gold Nanoparticles Enables Quantitative Detection of Attomolar Cancer Biomarkers. Anal. Chem..

[16] Qu W, Liu Y, Liu D, Wang Z, Jiang X (2011). Copper-mediated amplification allows readout of immunoassays by the naked eye. Angew. Chem. Int. Ed. Engl..

[17] Wang X, Niessner R, Knopp D (2015). Controlled growth of immunogold for amplified optical detection of aflatoxin B1. Analyst.

[18] Mie G (1908). Sättigungsstrom und Stromkurve einer schlecht leitenden Flüssigkeit. Ann. Phys..

[19] Prodan E, Radloff C, Halas NJ, Nordlander P (2003). A hybridization model for the plasmon response of complex nanostructures. Science.

[20] Fan, X., Zheng, W. & Singh, D. J. Light scattering and surface plasmons on small spherical particles. *Light. Sci. Appl*. **3** (2014).

[21] Schulz F (2014). Little Adjustments Significantly Improve the Turkevich Synthesis of Gold Nanoparticles. Langmuir.

[22] Yang Y, Shi J, Kawamura G, Nogami M (2008). Preparation of Au–Ag, Ag–Au core–shell bimetallic nanoparticles for surface-enhanced Raman scattering. Scr. Mater..

[23] Li JF (2010). Shell-isolated nanoparticle-enhanced Raman spectroscopy. Nature.

[24] Lyon JL, Fleming DA, Stone MB, Schiffer P, Williams ME (2004). Synthesis of Fe Oxide Core/Au Shell Nanoparticles by Iterative Hydroxylamine Seeding. Nano Lett..

[25] Kowalczyk B, Walker DA, Soh S, Grzybowski BA (2010). Nanoparticle supracrystals and layered supracrystals as chemical amplifiers. Angew. Chem. Int. Ed..

[26] Aili D, Selegård R, Baltzer L, Enander K, Liedberg B (2009). Colorimetric protein sensing by controlled assembly of gold nanoparticles functionalized with synthetic receptors. Small.

[27] Thanh NTK, Maclean N, Mahiddine S (2014). Mechanisms of nucleation and growth of nanoparticles in solution. Chem. Rev..

[28] Fu X, Chen L, Choo J (2017). Optical Nanoprobes for Ultrasensitive Immunoassay. Anal. Chem..

[29] Martínez-Aranguren R (2014). Is the Determination of Specific IgE against Components Using ISAC 112 a Reproducible Technique?. PLoS ONE.

