# Author Correction: Rapid signal enhancement method for nanoprobe-based biosensing

**DOI:** 10.1038/s41598-018-26155-4

**Published:** 2018-05-22

**Authors:** Jorge T. Dias, Gustav Svedberg, Mats Nystrand, Helene Andersson-Svahn, Jesper Gantelius

**Affiliations:** 1 Division of Proteomics and Nanobiotechnology, Science for Life Laboratory, KTH Royal Institute of Technology, Stockholm, Sweden; 2 Global Research and Development, Thermo Fisher Scientific IDD, Uppsala, Sweden

Correction to: *Scientific Reports* 10.1038/s41598-017-07030-0, published online 28 July 2017

In the Methods section of this Article references 18 to 22 are incorrectly cited. The correct references were omitted from the reference list and appear below as references 1–5. References 18 to 22 are correctly cited in Introduction and Results and Discussion sections.

“AuNPs of 10 nm in diameter were prepared following the protocol described by Bastús *et al*.^18^.”

should read:

“AuNPs of 10 nm in diameter were prepared following the protocol described by Bastús *et al*.^1^.”

“AgNPs of 90 nm in diameter were prepared following the protocol described by Rivero *et al*.^19^.”

should read:

“AgNPs of 90 nm in diameter were prepared following the protocol described by Rivero *et al*.^2^”

“The size was determined by UV-Vis spectroscopy according to the AgNPs size theory demonstrated by Malynych^20^.”

should read:

“The size was determined by UV-Vis spectroscopy according to the AgNPs size theory demonstrated by Malynych^3^.”

“The coupling of antibody to the NPs was prepared following a modified version of a protocol previously reported by Puertas *et al*.^21^.”

should read:

“The coupling of antibody to the NPs was prepared following a modified version of a protocol previously reported by Puertas *et al*.^4^.”

“Microarrays were prepared as previously reported by our group^22^.”

should read:

“Microarrays were prepared as previously reported by our group^5^.”
