# Supplementary material for: Rapid signal enhancement method for nanoprobe-based biosensing
Source: Sci Rep. 2017 Jul 28;7:6837. doi: 10.1038/s41598-017-07030-0 (PMC5533771; doi:10.1038/s41598-017-07030-0)
Supplement: Supplementary file 1 — Rapid signal enhancement method for nanoprobe-based biosensing SI [file 41598_2017_7030_MOESM1_ESM.pdf]

Rapid signal enhancement method for nanoprobe-based biosensing.

*Jorge T. Dias[1]; Gustav Svedberg[1]; Mats Nystrand[2]; Helene Andersson-Svahn[1]; Jesper Gantelius[1]**

[1]Division of Proteomics and Nanobiotechnology, Science for Life Laboratory, KTH Royal Institute of Technology, Sweden.[2] Global Research and Development, Thermo Fisher Scientific IDD, Uppsala, Sweden

Characterisation of the different sets of nanoparticles

- Transmission electron microscopy

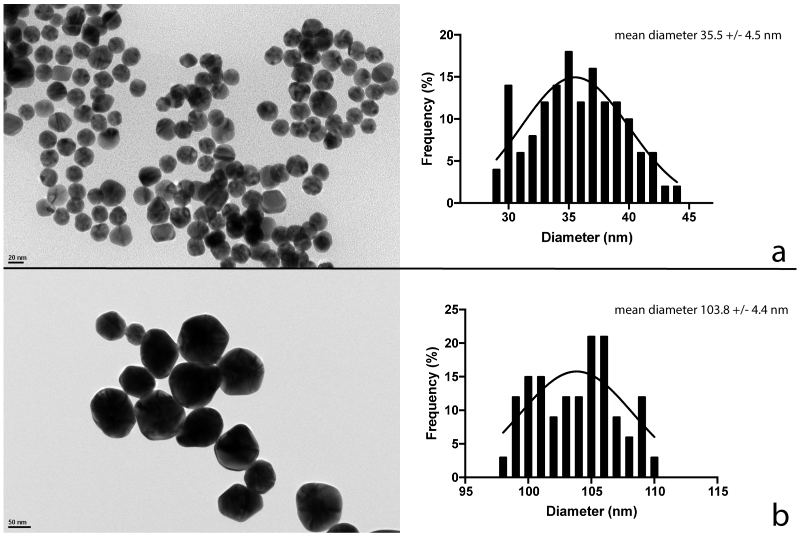

Figure S1. Transmission electron microscopy (TEM) micrographs of (**a**) AuNPs with a mean diameter of 35.5 nm and (**b**) AgNPs with a mean diameter of 103.8 nm. Histograms were obtained taking into account the diameter of a population of 150 NPs.

- Dynamic light scattering

Dynamic light scattering (DLS) measurements were performed on a Delsa Nano Beckman Coulter instrument at 25°C. Each sample was diluted in H_2O, and was measured three times, combining 10 runs per measurement (Figure S2).

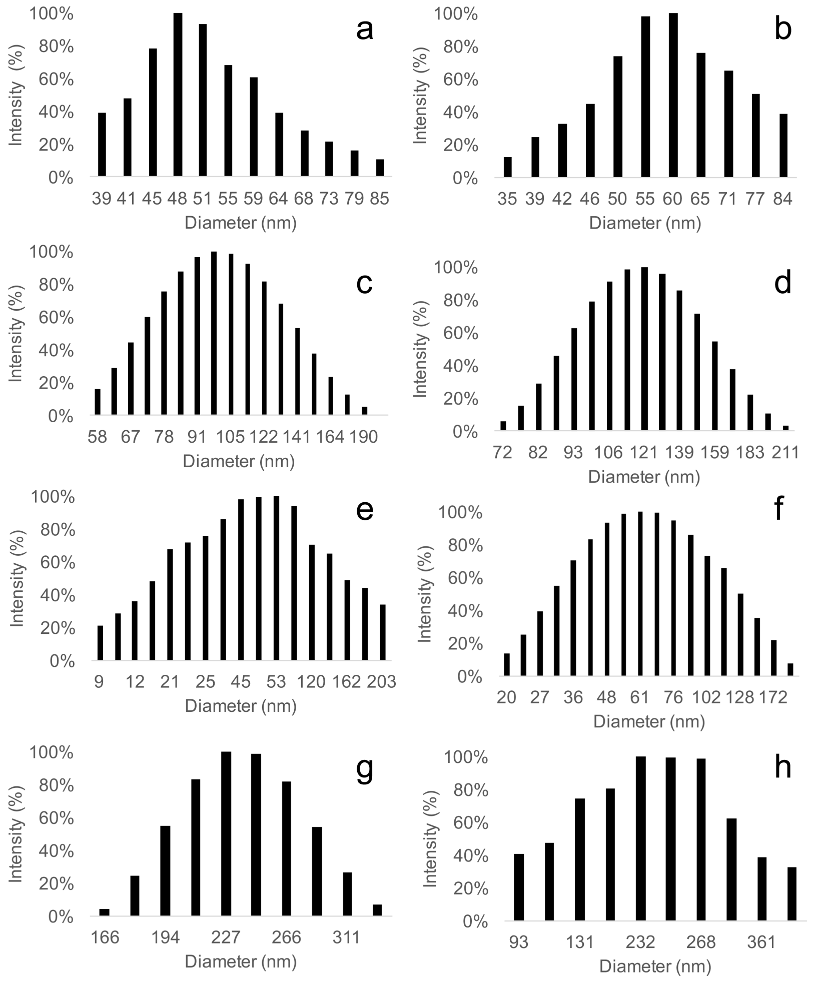

Figure S2. Hydrodynamic diameter of (**a**) AuNPs before surface modification with IgG; (**b**) AuNPs after surface modification with IgG. (**c**) AgNPs before surface modification with IgG; (**d**)AgNPs after surface modification with IgG. (**e**) SiNPs before modification with IgG; (**f**) SiNPs after modification with IgG. (**g**) IONPs before modification with IgG; (**h**) after modification with IgG. Each sample was diluted in water at pH 7 and was measured three times, combining 10 runs per measurement.

The effect on the diameter of the different sets of NPs was studied with dynamic light scattering (DLS). Prior the antibody modification, the AuNPs were modified with PEG-COOH molecules

(5 kDa), to provide anchor points for the antibody coupling. As observed by TEM, the synthesised AuNPs had an average diameter of 35 nm that shifted to approximately 50 nm after the decoration with PEG molecules (Figure S2a). The modification of these NPs with IgG resulted in their average diameter to increase to approximately 60 nm, as expected[1] (Figure S2b) Similarly, for the other sets of NPs an average increase of approximately 10 nm in diameter was observed after the modification with the IgG (Figure S2c-h)."

Characterisation and optimisation of enhancement solution

For gold growth to occur primarily in a defined region of the arrays where NPs are present it was required to optimize conditions for the reduction of Au^{3+} to Au^0. There are three main components involved in promoting the reduction of the gold salt: $HAuCl_4$ (gold precursor), H_2O_2 and MES buffer (reduction agents). The concentration of all three and the pH at which the reduction was allowed to occur was characterized. Optimising the system allowed gold colloid formation in solution prior to 5 minutes of incubation to be avoided since this would turn the solution from colourless to dark purple, compromising the signal/noise ratio. While spectrometric analysis was used to follow the arise of a SPR peak, reduction of $HAuCl_4$ could also be determined by observation of the colour change of the solution. From optimizations, conditions where no SPR peak was observed within 5 minutes were considered for further study. This time mark allowed us to assure that the conditions were favourable for gold nanoparticle formation while still being slow enough to favour the deposition of reduced Au^0 onto already existing nanoparticle seeds.

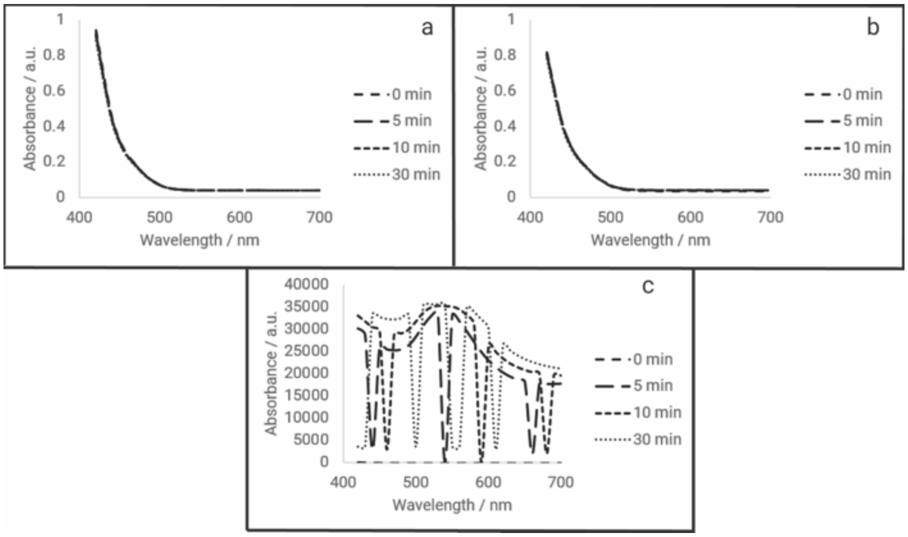

Figure S3: Uv-vis spectra of a solution containing 5mM HAuCl4, 10mM MES at pH 3 (**a**), 5mM HAuCl4, 10mM MES at pH 6 (**b**) and 5mM HAuCl4, 10mM MES at pH 11 (**c**) measured every 5 minutes for a total incubation time of 30 minutes.

Figure S3c shows that at pH 11 gold clusters start to form in solution. At pH 3 (Figure S3a) and pH 6 (Figure S3b) no SPR was detected, demonstrating that no gold nanoparticles were formed. Due to incompatibility with normal assay environments where the method could be applied, pH 11 conditions were not evaluated further.

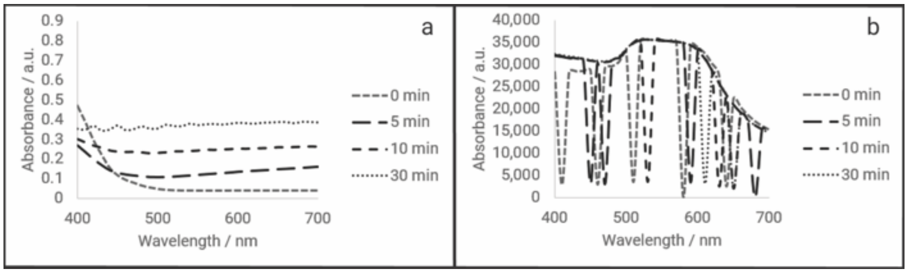

Figure S4: Uv-Vis spectra of a solution containing 5mM $HAuCl_4$, 6.035M H_2O_2, 10mM MES at pH 3 (**a**) and 5mM $HAuCl_4$, 6.035M H_2O_2, 10mM MES at pH 6 (**b**) measured every 5 minutes for a total incubation time of 30 minutes.

Figure S4 shows that for a concentration of 6.035 M H_2O_2 the formation of gold nanoparticles in solution started to occur at pH 3 (Figure S4a) and clearly occurred at pH 6 (Figure S4b).

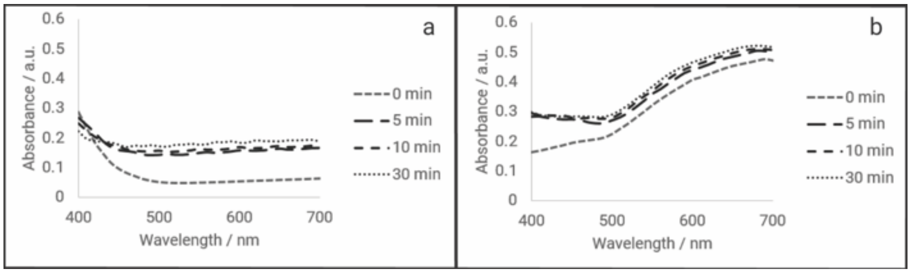

Figure S5: Uv-vis spectra of a solution containing 5mM $HAuCl_4$, 1.207M H_2O_2, 10mM MES at pH 3 (**a**) and 5mM $HAuCl_4$, 1.207M H_2O_2, 10mM MES at pH 6 (**b**) measured every 5 minutes until a total of 30 minutes of incubation was allowed.

By lowering the concentration of H_2O_2 to 1.027 M the time for formation of gold nanoparticles in solution was reduced as shown in figure S5.

The suggested enhancement effect was required to be rapid while minimizing the gold growth outside sensor regions already harboring nanoparticle detection probes. For proof of concept the following sets of conditions were allowed to incubate with a paper array where printed protein G spots had been detected by incubation with IgG-AuNPs (Figure S6).

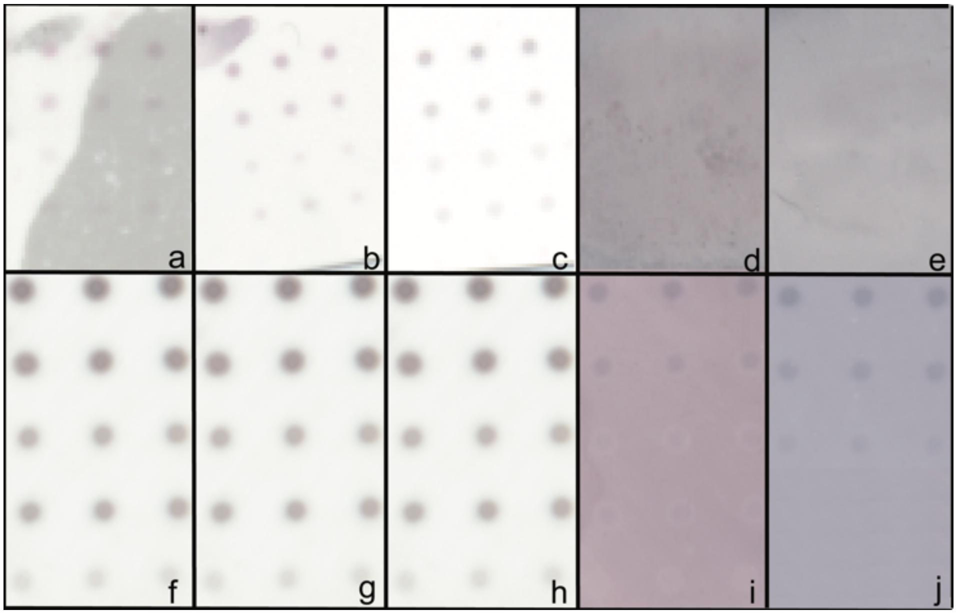

Figure S6. Vertical flow arrays after incubation with enhancement solution for 300 seconds. In each array, a gradient of Protein G molecules was printed, from top to bottom $2x10^7$, $2x10^6$, $2x10^5$, $2x10^4$, $2x10^3$ and $2x10^2$, respectively. After flowing IgG-AuNPs the arrays were allowed to incubate with (**a**) 50 mM MES pH 3, (**b**) 50 mM MES pH 4, (**c**) 50 mM MES pH 5, (**d**) 50 mM MES pH 6, (**e**) 50 mM MES

pH 7, (**f**) 50 mM MES pH 3, 1.027M H_2O_2, (**g**) 50 mM MES pH 4, 1.027M H_2O_2, (**h**) 50 mM MES pH 5, 1.027M H_2O_2, (**i**) 50 mM MES pH 6, 1.027M H_2O_2 and (**j**) 50 mM MES pH 7, 1.027M H_2O_2.

The change in colour from red to purple observed in the arrays was also observed when a solution of AuNPs was incubated with the enhancement solution (Figure S7). The SPR was observed to red-shift, characteristic of the formation of aggregates and/or increase in size of the nanoparticles. Although the absorbance intensity decreases with the formation of these larger, anisotropic nanoparticles, optically the intensity of the colour increases. This effect is due to the higher light scattering capability of these anisotropic nanoparticles.[2]

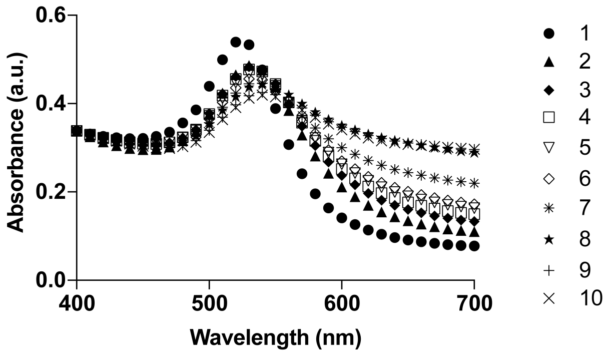

Figure S7. Absorbance spectra over a span of 10 minutes of a solution of AuNPs of 40 nm incubated with enhancement solution.

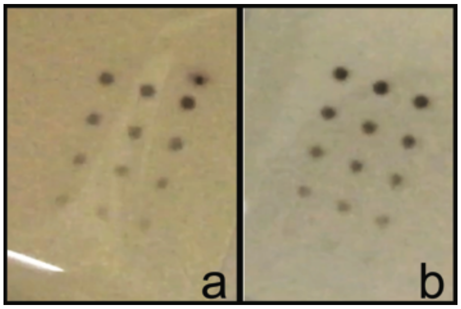

Figure S8. Video frame captured after 120 seconds into the enhancement method applied to arrays where IgG-modified AuNPs were allowed to detect a gradient concentration of plotted protein G. (**a**)

Enhancement solution consisting of 50mM MES pH 5 and 1.027M H_2O_2. (**b**) Enhancement solution consisting of 10mM MES pH 6 and 1.027M H_2O_2.

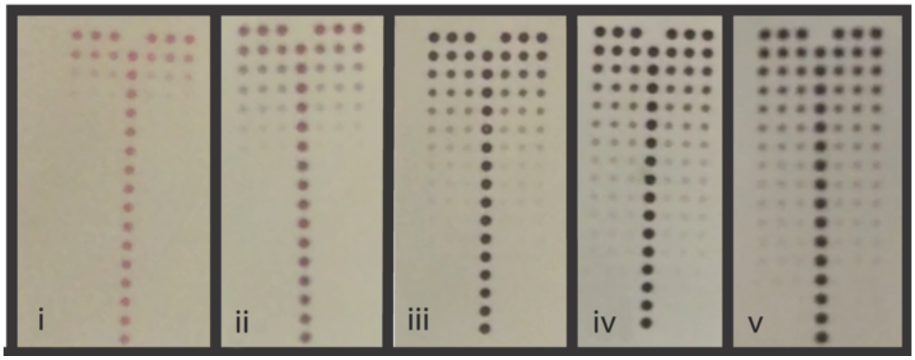

Figure S9. Spot intensity of printed AuNPs on paper (**i**) before incubation with enhancement solution; (**ii**) 60 seconds after incubation with enhancement solution; (**iii**) 120 seconds after incubation with enhancement solution; (**iv**) 210 seconds after enhancement solution and (**v**) 300 seconds after enhancement solution. Approximate number of AuNPs from top to bottom Lane 1 – AuNPs guideline; 2 – 100000 AuNPs; 3 – 30000 AUNPs; 4 – 10000 AuNPs; 5 – 3000 AuNPs; 6 – 1000 AuNPs; 7 – 300 AuNPs; 8 – 100 AuNPs; 9 – 75 AuNPs; 10 – 50 AuNPs; 11 – 25 AuNPs; 12 – 15 AuNPs; 13 – 10 AuNPs; 14 – 5 AuNPs; 15 – 2 AuNPs; 16 – 1 AuNP; 17 – BSA 1% for unspecific growth dismissal. On each paper strip, a T-shaped series of spots of high concentration AuNPs was deposited and used as a visual guide for localisation of the printed dilution series of AuNPs. Images were acquired with a 12-megapixel iSight camera (iPhone 6S).

Number of nanoparticles quantification

The volume and concentration of the gold nanoparticles' solution printed onto the paper support were taken into account for the estimation of the approximate number of nanoparticles.

The number of gold nanoparticles was calculated according to the following:

For a given nanoparticle radius r (cm), its volume was calculated with equation S1.

Equation S1: $$V = 4/3 * \pi * r^3$$

Knowing the density of gold ($g.cm^{-3}$), the mass (g) of each gold nanoparticle was calculated following equation S2:

Equation S2: nanoparticle mass (g) = density ($g.cm^{-3}$) * volume (cm^3)

The total number of nanoparticles for a given solution of nanoparticle's concentration was then calculated following equation S3:

Equation S3: total number of nanoparticles = solution of nanoparticle's concentration ($mg.mL^{-1}$) / nanoparticle mass (mg)

Linearity correlation prior and after enhancement.

Table 1. R^2 values for detection of protein G, prior and after enhancement, using different sets of nanoparticles.

	R^2 prior enhancement	R^2 after enhancement
IgG-AuNPs	$8.7x10^{-1}$	$7.8x10^{-1}$
IgG-AgNPs	$9.1x10^{-1}$	$9.6x10^{-1}$
IgG-IONPs	$9.17x10^{-1}$	$9.3x10^{-1}$
IgG-SiNPs	-	$8.5x10^{-1}$

Proof-of-concept with commercial IgE detection kit

Four serum samples were analysed with Thermo-Fischer's ImmunoCap ISAC array using fluorescent detection (ISAC-F) and using the presented gold enhancement detection (ISAC-G) to detect allergen-specific IgE.

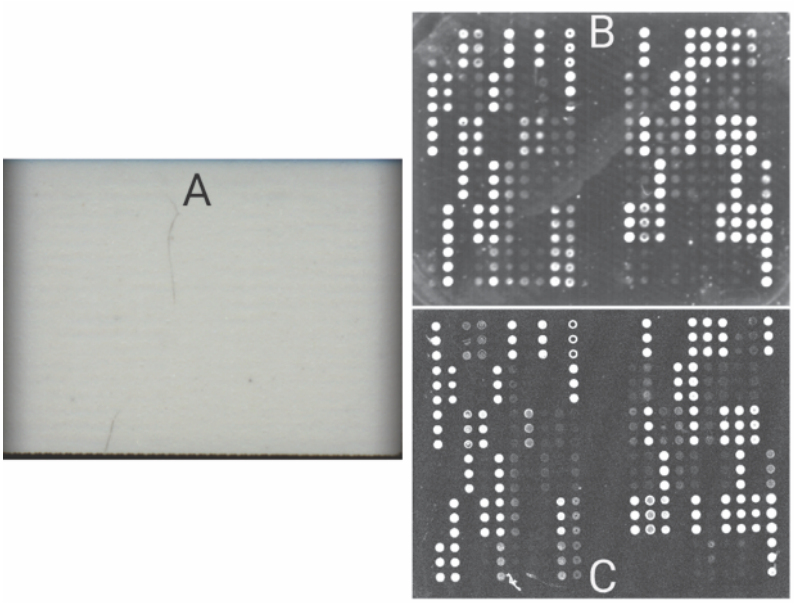

Figure S10: Scanned images of ISAC arrays used to detect allergen-specific IgE in a serum sample (**a**) prior to fluorescence or gold enhancement, (**b**) using fluorescent detection (**c**) and using the presented gold enhancement detection. Allergens are deposited in vertical triplicates with positive controls for fluorescent detection on the far-right bottom. (The other three corners are used for software evaluation) In the fluorescence-based detection, the brightness of spots is fluorescence emission, whereas in the gold enhancement-based detection, the image has been inverted and increased brightness indicates a colourimetrically darker spot.

References

1. Filipe, V., Hawe, A. & Jiskoot, W. Critical evaluation of Nanoparticle Tracking Analysis (NTA) by NanoSight for the measurement of nanoparticles and protein aggregates. *Pharm. Res.* **27,** 796–810 (2010).
2. Fan, X., Shen, Z. & Luk'yanchuk, B. Huge light scattering from active anisotropic spherical particles. *Opt. Express* **18,** 24868–24880 (2010).
